# Zolpidem Use and the Risk of Injury: A Population-Based Follow-Up Study

**DOI:** 10.1371/journal.pone.0067459

**Published:** 2013-06-27

**Authors:** Shiu-Dong Chung, Ching-Chun Lin, Li-Hsuan Wang, Herng-Ching Lin, Jiunn-Horng Kang

**Affiliations:** 1 Division of Urology, Department of Surgery, Far Eastern Memorial Hospital, New Taipei City, Taiwan; 2 Sleep Research Center, Taipei Medical University Hospital, Taipei, Taiwan; 3 Graduate Institute of Biomedical Informatics, Taipei Medical University, Taipei, Taiwan; 4 School of Pharmacy, Taipei Medical University, Taipei, Taiwan; 5 Department of Pharmacy, Taipei Medical University Hospital, Taipei, Taiwan; 6 Department of Physical Medicine and Rehabilitation, Taipei Medical University Hospital, Taipei, Taiwan; 7 Department of Physical Medicine and Rehabilitation, School of Medicine, College of Medicine, Taipei Medical University, Taipei, Taiwan; Hôpital Robert Debré, France

## Abstract

**Background:**

While an association between zolpidem use and fracture and road accident was previously proposed, this study aimed to further explore the frequency and risk of a wide spectrum of injuries in subjects prescribed with zolpidem in Taiwan.

**Methods:**

We identified 77,036 subjects who received Zolpidem treatment between 2005 and 2007. We randomly selected 77,036 comparison subjects who were frequency-matched based-on their demographic profiles. We individually tracked each subject for a 90-day period to identify those who subsequently suffered an injury. Cox proportional hazards regressions were performed to calculate the hazard ratio of injury between the two groups.

**Results:**

The incidence rate of injury during the 90-day follow-up period for the total subjects was 18.11 (95% CI = 17.69–18.54) per 100 person-years; this was 24.35 (95% CI = 23.66–25.05) and 11.86 (95% CI = 11.39–12.36) for the study and comparison cohort, respectively. After adjusting for demographic variables, the hazard ratio (HR) of injury during the 90-day follow-up period for study subjects was 1.83 (95% CI = 1.73–1.94) that of comparison subjects. Additionally, compared to comparison subjects, the adjusted HR of injury during the 90-day follow-up period for study subjects who were prescribed Zolpidem for >30 days was as high as 2.17 (95% CI = 2.05–2.32). The adjusted HR of injury to blood vessels for study subjects was particularly high when compared to comparison subjects (HR = 6.34; 95% CI = 1.37–29.38).

**Conclusions:**

We found that patients prescribed with Zolpidem were at a higher risk for a wide range of injuries.

## Introduction

Zolpidem is a short-acting non-benzodiazepine drug used to treat insomnia. The onset of zolpidem is fast, which is suitable for subjects who have difficulty initiating sleep [1]. However, by interacting with GABA-a receptor, zolpidem exhibits common central nervous system side effects similar to benzodiazepine-type hypnotics including drowsiness, dizziness, and impaired posture control and coordination, etc [1]. The use of benzodiazepines has been considered to increase the risk for falls and hip fractures, particularly among the elderly [2]–[5]. Furthermore, the use of these medications may impair reaction time and driving skills, hence these medications may also increase the risk of road traffic accidents [6], [7].

Because zolpidem is fast-acting and has a short half-life (2 hours) in the body, users generally experience a relatively small residual effect the following day compared with longer-acting benzodiazepines. Although still controversial, zolpidem is generally regarded to be a safer medication for treating insomnia [1], [8]. However, it is increasingly recognized that some parasomnia, such as sleep-eating, sleep-walking, and sleep-driving occurs among some patients taking zolpidem [9]. These unconscious behaviors during sleep may cause severe self-injury and raise concerns regarding the safety of zolpidem. In addition, several studies have also reported that an increased risk for fracture and road accidents can still be found among subjects who used zolpidem [10], [11].

As zolpidem is commonly prescribed, and is by far the most widely prescribed non-benodiazepine hypnotic for insomnia in Taiwan, the safety of its use is an important public health issue. Previous studies have mainly focused on the risk of fracture and road accidents in the patients who used zolpidem, and may have underestimated the overall impact of the drug regarding its association with the occurrence of injury. Clinical guidelines should be developed based on epidemiological evidence of this potential association. Hence, the present study aimed to explore the profile of a wide spectrum of injury occurrence among cohort of patients prescribed with zolpidem compared with a matched comparison cohort using a large population-based retrospective database.

## Methods

### Database

This retrospective cohort study used data sourced from the Longitudinal Health Insurance Database 2000 (LHID2000). The LHID2000 consists of the original claims data and registration files for 1,000,000 enrollees under the Taiwan National Health Insurance (NHI) program. Taiwan Initiated the NHI program in March 1995. The 1,000,000 subjects included in the LHID2000 were randomly sampled from the 2000 Registry for Beneficiaries of the NHI program. Studies have demonstrated the high validity of the data from the NHI program [12], [13]. Many researchers have employed the LHID2000 to perform and publish their studies in internationally peer-reviewed journals [14].

As the LHID2000 consists of de-identified secondary data released to the public for research purposes, after consulting with the director of the Institutional Review Board (IRB), this study was exempted from full review and approved by the Taipei Medical University IRB.

### Sample Selection

This study featured a study cohort and comparison cohort. For the study cohort, we identified 80,351 subjects aged ≥18 years that were prescribed with Zolpidem between January 2005 and December 2007. We assigned the date of their first Zolpidem prescription as their index date and excluded all of the subjects that had suffered any of the injuries selected for this analysis within the 90 days (*n* = 2,495) preceding their index dates or had received Zolpidem treatment within one year prior to their index date. In addition, we excluded all the subjects who had a history of epilepsy (ICD-9-CM code 345) or infantile cerebral palsy (ICD-9-CM code 343) (n = 820). Ultimately, 77,036 subjects were included in the study cohort.

We likewise selected comparison subjects from the remaining enrollees of the LHID2000. A total of 77,036 comparison subjects were randomly selected and frequency-matched with subjects in the study cohort by sex, age group (18–29, 30–29, 40–49, 50–59, 60–69, 70–79, and >79), urbanization level, and index year. We selected the urbanization level of the subject’s residence as a matching criterion in order to help control for error variables, namely unmeasured neighborhood socioeconomic characteristics between the study and comparison cohort. For comparison subjects, we assigned their first use of medical care occurring during the index year as their index date. We assured that none of the selected comparison subjects were prescribed Zolpidem treatment within one year following or prior to their index date. In addition, we assured that none of the selected comparison subjects had a history of epilepsy or infantile cerebral palsy.

Furthermore, according to the formula demonstrated by Hsieh and Lavori [15], the required number of events for a proportional hazards regression in this study is calculated to be 676 under the condition of significance level and type II error setting to be 0.05 and 0.05, respectively. Therefore, the sample size included in this study has enough statistical power to detect the statistically significant difference in the risk of injury between the study cohort and the comparison cohort.

### Variables of Interest

We individually tracked 154,072 subjects for a 90-day period starting from their index date to identify those subjects who subsequently received a diagnosis of injury. We classified the types of injury into the following categories: fracture (ICD-9-CM codes 800-829), dislocation (ICD-9-CM codes 830-839), sprain (ICD-9-CM codes 840-848), intracranial injury (ICD-9-CM codes 850-854), internal injury of thorax and pelvis (ICD-9-CM codes 860-869), open wound (ICD-9-CM codes 870-897), injury to blood vessels (ICD-9-CM codes 900-904), burns (ICD-9-CM codes 940-949), injury to nerves and spinal cord (ICD-9-CM codes 950-957), and injury, other and unspecified (ICD-9-CM codes 959). Furthermore, this study also analyzed the risk of injury according to the period of time patients received Zolpidem treatment (≤30 days and >30 days).

### Statistical Analysis

We used the SAS statistical package to perform all the statistical analyses performed in this study. We used the Kaplan-Meier method to estimate one-year injury-free survival rates and used the log-rank test to examine differences in injury-free survival rates between the study and comparison cohort. Furthermore, stratified Cox proportional hazards regressions (stratified on sex, age, urbanization level, and the year of index date) were carried out to explore difference in 90-day injury-free survival rates between the study and comparison cohort. We adjusted for patient’s co-morbidities by using the Elixhauser Co-morbidity Index, which was created in 1997 and uses 30 binary (1 = present and 0 = absent) co-morbidity measures to account for patient morbidity and mortality. In addition, we calculated a propensity score for each subjects and adjusted for propensity in all regression models. A propensity score was initially used to balance demographic and treatment characteristics, which were distributed unequally between the study cohort and the comparison cohort. Because the probability of injury may depend on the subject’s age, sex, urbanization level, monthly income, prior history of injury within one year prior to index date, and medical co-morbidities, were entered into a multivariable logistic regression model as predictors, to calculate the expected probability of injury for each subject. A two-sided *p*-value of <0.05 was considered statistically significant for this study.

## Results

The distribution of demographic characteristics and co-morbidities between the study cohort and comparison cohort was presented in Table 1. Of the 154,072 subjects, about 61% were females and 25.1% were aged less than 40 years. After matching for sex, age group, and urbanization level, there was no significant difference in the distribution of monthly income and geographic region between the study and comparison cohort. The study cohort had a higher prevalence of all selected co-morbidities than the comparison cohort except for AIDS.

**Table 1:** Comparisons of sociodemographic characteristics of subjects who were prescribed Zolpidem and comparison subjects, 2005–2007 (n = 154,072).

Variable	Subjects who were prescribed Zolpidem (*n* = *77,036*)		Comparison subjects (*n = 77,036*)		P value
	Total No.	%	Total No.	%	
Sex					1.000
Male	29,810	38.7	29,810	38.7	
Female	47,226	61.3	47,226	61.3	
Age (years)					1.000
18–29	8,039	10.4	8,039	10.4	
30–39	11,300	14.7	11,300	14.7	
40–49	15,410	20.0	15,410	20.0	
50–59	16,039	20.8	16,039	20.8	
60–69	11,399	14.8	11,399	14.8	
70–79	10,229	13.3	10,229	13.3	
>79	4,620	6.0	4,620	6.0	
Monthly income					0.271
≤ NT15,840	34,894	45.3	34,655	45.0	
NT$15,841-25,000	26,803	34.8	26,807	34.8	
≥ NT$25,001	15,339	19.9	15,574	20.2	
Urbanization level					1.000
1 (most urbanized)	22,382	29.1	22,382	29.1	
2	21,984	28.5	21,984	28.5	
3	11,911	15.5	11,911	15.5	
4	11,218	14.5	11,218	14.5	
5 (least urbanized)	9,541	12.4	9,541	12.4	
Geographic region					0.256
Northern	35,963	46.7	35,618	46.2	
Central	20,262	26.3	20,600	26.7	
Southern	18,375	23.8	18,262	23.7	
Eastern	2,436	3.2	2,556	3.4	
Cardiac arrhythmias	9,911	12.9	4,745	6.2	<0.001
Congestive heart failure	6,314	8.2	3,110	4.0	<0.001
Valvular disease	1,926	2.5	847	1.1	<0.001
Pulmonary circulation disorders	157	0.2	71	0.1	<0.001
Periphreal vascular disorders	4,202	5.5	2,175	2.8	<0.001
Hypertension	33,683	43.7	22,924	29.8	<0.001
Paralysis	2,422	3.1	1,454	1.9	<0.001
Coagulopathy	816	1.1	461	0.6	<0.001
Other neurological disorders	4,622	6.0	1,878	2.4	<0.001
Chronic pulmonary disease	19,298	25.1	11,763	15.3	<0.001
Diabetes, uncomplicated	15,222	19.8	9,996	13.0	<0.001
Diabetes, complicated	7,099	9.2	4,099	5.3	<0.001
Hypothyroidism	8,119	10.5	4,764	6.2	<0.001
Renal failure	3,251	4.2	1,581	2.1	<0.001
Liver disease	18,317	23.8	11,314	14.7	<0.001
Peptic ulcer disease excluding bleeding	23,331	30.3	13,010	16.9	<0.001
Solid tumor without metastatsis	6,118	7.9	3,155	4.1	<0.001
Rheumatoid arthritis	6,944	9.0	3,885	5.0	<0.001
Fluid and electrolyte disorders	3,453	4.5	1,288	1.7	<0.001
Blood loss anemia	497	0.7	314	0.4	<0.001
Deficiency anemias	2,157	2.8	1,156	1.5	<0.001
Alcohol abuse	1,435	1.9	219	0.3	<0.001
Drug abuse	3,316	4.3	1,174	1.5	<0.001
Psychoses	11,548	15.0	1,452	1.9	<0.001
Depression	16,676	21.7	2,130	2.8	<0.001
AIDS	60	0.0	52	0.0	0.449
Lymphoma	1,695	2.2	847	1.1	<0.001
Metastatic cancer	554	0.7	165	0.2	<0.001
Obesity	966	1.3	554	0.7	<0.001
Weight loss	462	0.6	257	0.3	<0.001

**Figure 1:**
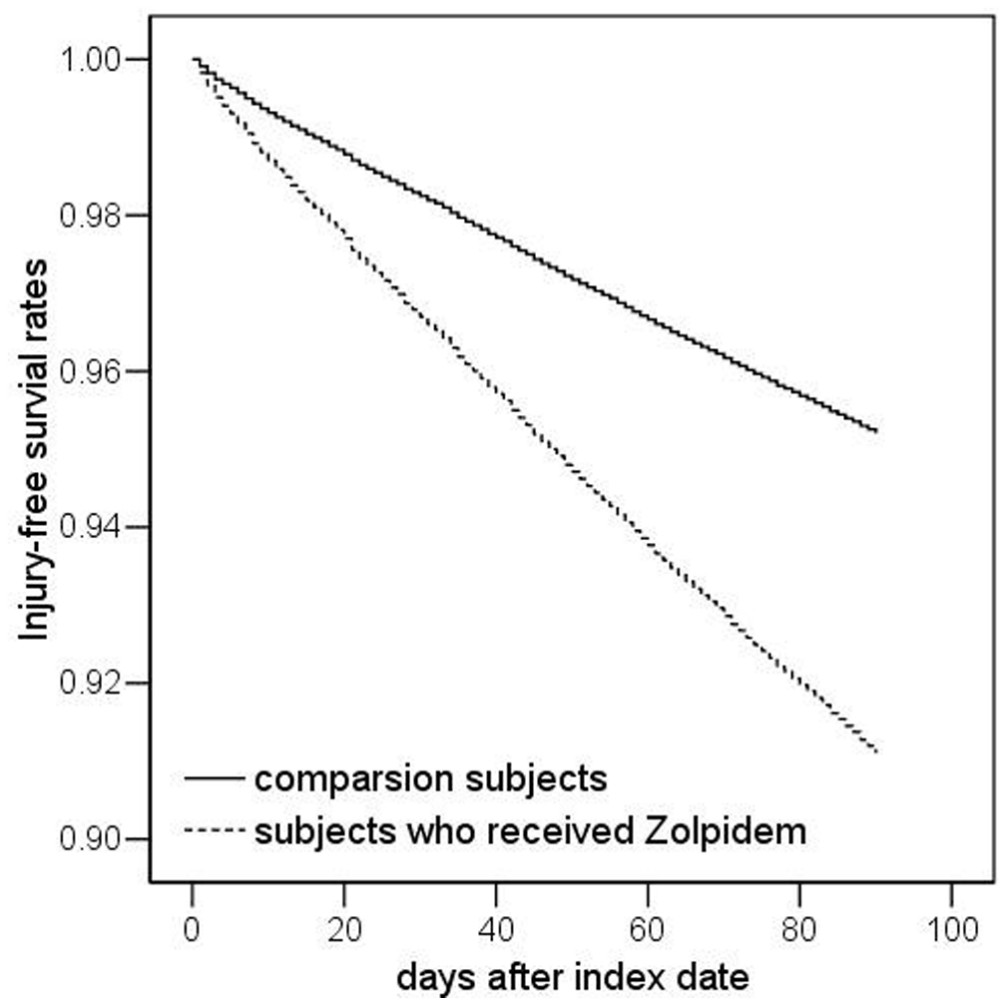
Injury-free survival rates for subjects who were prescribed Zolpidem treatment and the comparison subjects.

Table 2 shows the incidence of injury during the 90-day follow-up period of the sampled subjects. The incidence rate of injury during the 90-day follow-up period for the total sampled subjects was 18.11 (95% CI = 17.69–18.54) per 100 person-years; this was 24.35 (95% CI = 23.66–25.05) and 11.86 (95% CI = 11.39–12.36) for the study and comparison cohort, respectively. The log-rank test also suggested that study subjects had a significantly lower 90-day injury-free survival rate than comparison subjects (*p*<0.001). Figure 1 presented the Kaplan-Meier method injury-free survival curves for study and comparison subjects.

**Table 2:** Incidence rate, crude and adjusted hazard ratios for injury among the sampled subjects.

Presence of injury during the 90-day follow-up period	Total *(n = 154,072*)		Subjects who were prescribed Zolpidem (*n* = *77,036*)		Comparison subjects (*n = 77,036*)	
	*n*, %		*n*, %		*n*, %	
Injury	6,974	4.5	4,689	6.1	2,285	3.0
Incidence rate per 100 person-years (95% CI)	18.11 (17.69–18.54)		24.35 (23.66–25.05)		11.86 (11.39–12.36)	
Crude HR^a^ (95% CI)	–		2.12*** (2.02–2.23)		1.00	
Adjusted HR^b^ (95% CI)	–		1.83*** (1.73–1.94)		1.00	

*Notes:* ***indicates *p*<0.001.

a Hazard ratio was calculated by using stratified Cox proportional regression (stratified on sex and age group).

b Adjustment for patient’s monthly income, urbanization level, geographic region, cardiac arrhythmias, congestive heart failure, valvular disease, pulmonary circulation disorders, periphreal vascular disorders, hypertension, paralysis, coagulopathy, other neurological disorders, chronic pulmonary disease, diabetes uncomplicated, diabetes complicated, hypothyroidism, renal failure, liver disease, peptic ulcer disease excluding bleeding, solid tumor without metastatsis, rheumatoid arthritis, fluid and electrolyte disorders, blood loss anemia, deficiency anemias, alcohol abuse, drug abuse, psychoses, depression, AIDS, lymphoma, metastatic cancer, obesity, weight loss, and propensity score.

The hazard ratio (HR) of injury between study and comparison subjects was also presented in Table 2. After adjusting for geographic region, monthly income, co-morbidities, and propensity score, the HR of injury during the 90-day follow-up period for study subjects was 1.83 (95% CI = 1.73–1.94) that of comparison subjects.

We further analyzed the risk of injury by the length of Zolpidem treatment. Table 3 shows that compared to comparison subjects, the adjusted HR of injury during the 90-day follow-up period for study subjects who were prescribed Zolpidem for >30 days was as high as 2.17 (95% CI = 2.05–2.32). The adjusted HR of injury for the study subjects who were prescribed Zolpidem for ≤30 days was 1.55 (95% CI = 1.45–1.66) that of the comparison cohort.

**Table 3:** Incidence rate, crude and adjusted hazard ratios for injury among the sampled subjects, by the length of Zolpidem treatment.

Presence of Injury during the 90-day follow-up period	Comparison subjects (*n = 77,036*)		Subjects who were prescribed Zolpidem (*n* = *77,036*)			
			≤30 days (*n = 36,528*)		>30 days (*n = 40,508*)	
	*n*, %		*n*, %		*n*, %	
Injury	2,285	3.0	1,769	4.8	2,920	7.2
Incidence rate per 100 person-years (95% CI)	11.86 (11.39–12.36)		19.37 (18.48–20.29)		28.83 (27.80–29.89)	
Crude HR^a^ (95% CI)	1.00		1.67*** (1.56–1.77)		2.54*** (2.40–2.69)	
Adjusted HR^b^ (95% CI)	1.00		1.55*** (1.45–1.66)		2.17*** (2.05–2.32)	

*Notes:* *** indicates *p*<0.001.

a Hazard ratio was calculated by using stratified Cox proportional regression (stratified on sex and age group).

b Adjustment for patient’s monthly income, urbanization level, geographic region, cardiac arrhythmias, congestive heart failure, valvular disease, pulmonary circulation disorders, periphreal vascular disorders, hypertension, paralysis, coagulopathy, other neurological disorders, chronic pulmonary disease, diabetes uncomplicated, diabetes complicated, hypothyroidism, renal failure, liver disease, peptic ulcer disease excluding bleeding, solid tumor without metastatsis, rheumatoid arthritis, fluid and electrolyte disorders, blood loss anemia, deficiency anemias, alcohol abuse, drug abuse, psychoses, depression, AIDS, lymphoma, metastatic cancer, obesity, weight loss, and propensity score.

Table 4 shows the HR of injury stratified by age group. It shows that study subjects had a consistently and significantly higher HR of injury during the 90-day follow-up period than comparison cohort across all age groups.

**Table 4:** Incidence rate, crude and adjusted hazard ratios for injury among the sampled subjects by age group.

Presence of Injury during the 90-day follow-up period	Age group					
	18–39		40–59		>59	
	Subjects who were prescribed Zolpidem	Comparison subjects	Patients erectile dysfunction	Controls	Patients erectile dysfunction	Controls
			*n*, %	*n*, %	*n*, %	*n*, %
Yes	1,161 (6.0)	517 (2.7)	1,821 (5.8)	921 (2.9)	1,707 (6.5)	847 (3.2)
Crude HR^a^ (95% CI)	2.32*** (2.09–2.58)	1.00	2.04*** (1.88–2.21)	1.00	2.09*** (1.92–2.27)	1.00
Adjusted HR^b^ (95% CI)	1.91*** (1.69–2.14)	1.00	1.75*** (1.60–1.92)	1.00	1.86*** (1.71–2.05)	1.00

*Notes:* *** indicates *p*<0.001.

a Hazard ratio was calculated by using stratified Cox proportional regression (stratified on sex and age group).

b Adjustment for patient’s monthly income, urbanization level, geographic region, cardiac arrhythmias, congestive heart failure, valvular disease, pulmonary circulation disorders, periphreal vascular disorders, hypertension, paralysis, coagulopathy, other neurological disorders, chronic pulmonary disease, diabetes uncomplicated, diabetes complicated, hypothyroidism, renal failure, liver disease, peptic ulcer disease excluding bleeding, solid tumor without metastatsis, rheumatoid arthritis, fluid and electrolyte disorders, blood loss anemia, deficiency anemias, alcohol abuse, drug abuse, psychoses, depression, AIDS, lymphoma, metastatic cancer, obesity, weight loss, and propensity score.

Furthermore, we analyzed the HR of injury according to injury type (Table 5). We found that study subjects had consistently higher risks of injury than comparison subjects across different types of injury. The adjusted HR of injury to blood vessels for study subjects was particularly high when compared to comparison subjects (HR = 6.34; 95% CI = 1.37–29.38). In addition, the adjusted HR of intracranial injury among study subjects was 2.39 (95% CI = 1.81–3.14) that of comparison subjects.

**Table 5:** Incidence rate, crude and adjusted hazard ratios for injury among the sampled subjects according to injury type.

Presence of Injury during the 90-day follow-up period	Total *(n = 154,072*)					Subjects who were prescribed Zolpidem (*n* = *77,036*)					Comparison subjects (*n = 77,036*)			
	*n*, %					*n*, %					*n*, %			
Fracture	752			0.49		520			0.68		232		0.30	
Crude HR^a^ (95% CI)	–					2.25*** (1.93–2.63)					1.00			
Adjusted HR^b^ (95% CI)	–					1.79*** (1.51–2.10)					1.00			
Dislocation	59			0.04		40			0.05		19		0.02	
Crude HR^a^ (95% CI)	–					2.11** (1.22–3.64)					1.00			
Adjusted HR^b^ (95% CI)	–					1.61 (0.90–2.91)					1.00			
Sprain	3,826			2.48		2,534			3.29		1,292		1.68	
Crude HR^a^ (95% CI)	–					1.99*** (1.86–2.13)					1.00			
Adjusted HR^b^ (95% CI)	–					1.83*** (1.70–1.97)					1.00			
Intracranial injury	314			0.20		235			0.31		79		0.10	
Crude HR^a^ (95% CI)	–					2.98*** (2.31–3.85)					1.00			
Adjusted HR^b^ (95% CI)	–					2.39*** (1.81–3.14)					1.00			
Internal injury of thorax, abdomen, and pelvis	25			0.02		19			0.02		6		0.01	
Crude HR^a^ (95% CI)	–					3.17* (1.27–7.93)					1.00			
Adjusted HR^b^ (95% CI)	–					2.46 (0.92–6.57)					1.00			
Open wounds	1,824			1.18		1,226			1.59		598		0.78	
Crude HR^a^ (95% CI)	–					2.07*** (1.87–2.28)					1.00			
Adjusted HR^b^ (95% CI)	–					1.75*** (1.57–1.93)					1.00			
Injury to blood vessels	15			0.01		13			0.02		2		0.00	
Crude HR^a^ (95% CI)	–					6.50* (1.47–28.81)					1.00			
Adjusted HR^b^ (95% CI)	–					6.34* (1.37–29.38)					1.00			
Burns	201			0.13		135			0.18		66		0.09	
Crude HR^a^ (95% CI)	–					2.05*** (1.53–2.75)					1.00			
Adjusted HR^b^ (95% CI)	–					1.78*** (1.29–2.46)					1.00			
Injury to nerves and spinal cord	55		0.04			42			0.05		13		0.02	
Crude HR^a^ (95% CI)	–					3.23*** (1.74–6.02)					1.00			
Adjusted HR^b^ (95% CI)	–					2.06* (1.06–4.02)					1.00			
Injury, other and unspecified	170			0.11		108			0.14		62		0.08	
Crude HR^a^ (95% CI)	–					1.74*** (1.28–2.38)					1.00			
Adjusted HR^b^ (95% CI)	–					1.25 (0.88–1.77)					1.00			

*Notes:* *indicates *p*<0.05; **indicates *p*<0.01;***indicates *p*<0.001; ^a^Hazard ratio was calculated by using stratified Cox proportional regression (stratified on sex and age group); ^b^Adjustment for patient’s monthly income, urbanization level, geographic region, cardiac arrhythmias, congestive heart failure, valvular disease, pulmonary circulation disorders, periphreal vascular disorders, hypertension, paralysis, coagulopathy, other neurological disorders, chronic pulmonary disease, diabetes uncomplicated, diabetes complicated, hypothyroidism, renal failure, liver disease, peptic ulcer disease excluding bleeding, solid tumor without metastatsis, rheumatoid arthritis, fluid and electrolyte disorders, blood loss anemia, deficiency anemias, alcohol abuse, drug abuse, psychoses, depression, AIDS, lymphoma, metastatic cancer, obesity, weight loss, and propensity score.

Table 6 presents the HR of injury during the 90-day follow-up period after terminating the use of Zolpidem. It shows that the study subjects still had a higher HR for injury than the comparison cohort after terminating the use of Zolpidem. However, the magnitude of association was significantly attenuated.

**Table 6:** Incidence rate, crude and adjusted hazard ratios for injury after terminating the use of Zolpidem among the sampled subjects.

Presence of injury during the 90-day follow-up period after terminating the use of Zolpidem	Comparison subjects (*n = 77,036*)		Subjects who were prescribed Zolpidem (*n* = *77,036*)			
			≤30 days (*n = 36,528*)		>30 days (*n = 40,508*)	
	*n*, %		*n*, %		*n*, %	
Injury	2,285	3.0	1,315	3.6	1,791	4.4
Crude HR^a^ (95% CI)	1.00		1.22*** (1.14–1.31)		1.69***(1.59–1.80)	
Adjusted HR^b^ (95% CI)	1.00		1.14** (1.06–1.22)		1.52***(1.43–1.62)	

*Notes:* **indicates *p*<0.01;***indicates *p*<0.001; ^a^Hazard ratio was calculated by using stratified Cox proportional regression (stratified on sex and age group); ^b^Adjustment for patient’s monthly income, urbanization level, geographic region, cardiac arrhythmias, congestive heart failure, valvular disease, pulmonary circulation disorders, periphreal vascular disorders, hypertension, paralysis, coagulopathy, other neurological disorders, chronic pulmonary disease, diabetes uncomplicated, diabetes complicated, hypothyroidism, renal failure, liver disease, peptic ulcer disease excluding bleeding, solid tumor without metastatsis, rheumatoid arthritis, fluid and electrolyte disorders, blood loss anemia, deficiency anemias, alcohol abuse, drug abuse, psychoses, depression, AIDS, lymphoma, metastatic cancer, obesity, weight loss, and propensity score.

## Discussion

We found that patients who had received zolpidem treatment were at a higher risk (1.96 times) for the occurrence of injury than a matched population of comparison patients. This increased risk was uniformly detected across a wide range of injuries. The mechanism of this association is still unclear. Nevertheless, the association between zolpidem and injury should not be simplified to a direct-causality relationship in the interpretation of our results. Similar to benzodiazepine, previous studies have reported that patients treated with zolpidem may be at higher risk for falls and road traffic accidents [10], [11]. Therefore, the elevated risk for injury among patients receiving treatment with zolpidem should not be overlooked. It should also be emphasized that individual susceptibility to the side effect of zolpidem may be modified by patient age, general condition, comorbidity and co-used medications. A comprehensive and careful evaluation should be conducted before zolpidem is prescribed to minimize the risk for potential injury. Although zolpidem has some favorable pharmacokinetic characteristics in treating sleep initiation difficulties related to insomnia, both physicians and patients should be aware of the association between zolpidem and the potential risk for injury.

To the best of our knowledge, previous reports regarding the risk for these specific forms of injury remain scanty. In our study, it is worth noting that there was an increased frequency of severe forms of injury, including intracranial injury (HR = 2.39), internal visceral injury (HR = 2.46), and injury to the nerves and spinal cord (HR = 2.06). This form of injury is often associated with relatively high-energy impact/collision such as road traffic accidents, falling from heights, and other industrial accidents. Intracranial injury and spinal cord injury can cause significant neurological deficits and are associated with high mortality and long-term morbidity and disability. In addition, these injuries can cause significant medical and economic burdens. Previous studies reported psychoactive drug and alcohol usage can be associated with fatal road traffic accident [16], [17]. However, the current literature lacks data regarding a detailed injury spectrum in this population.

The dose-response association between zolpidem and injury remains controversial. Some authors noted no clear dose-response association between short-acting benzodiazepine and fracture [4]. In the present study, we did not analyze the daily dose of zolpidem since the NHI restricts the use of zolpidem to 10 mg per day. However, we did find that patients who were prescribed with zolpidem for a longer duration (>30 days) were at higher risk for the occurrence of injury than those patients prescribed for a shorter duration (<30 days). Since zolpidem has a short half-life, this finding is difficult to be understood by explanations based-on accumulation of the drug. Nevertheless, this finding may be partially confounded by the severity of underlying insomnia and psychological disorders. There is also still limited data concerning the safety of long-term zolpidem use, and future studies aimed at evaluating the long-term safety of zolpidem use are advised to take the occurrence of injury into consideration.

We found that patients receiving zolpidem treatment were at higher risk for fracture than matched a cohort. This finding is consistent with one previous study. Finkle et al. reported an excessive risk comparable with diazepam for non-vertebral-fracture and dislocation requiring hospitalization among patients treated with zolpidem [11]. Contrary to these results, Chang et al. found the use of zolpidem to not be associated with a higher risk of hip fracture in a population restricted to those over the age of 65 years [3]. This discrepancy regarding the risk for fracture may be due to methodological differences such as inclusion criteria, comparison groups, and adjustment variables.

The most important issue in exploring the risk of zolpidem and injury occurrence is the confounding effect of underlying insomnia. Insomnia can impair daytime function and psychological status, and therefore is likely to increase the risk for accident, fall and injury [18]. Although some epidemiological studies have utilized self-comparisons regarding the risk of fall between pre-treatment and post-treatment of medication, the confounding effects of insomnia cannot be excluded in this design. However, we have analyzed for the risk of injury during the 90 days following termination of Zolpidem use (Table 6). Since the risk following use was significantly attenuated in comparison to the during use estimate, we feel that this provides evidence supporting that the drug had an effect on the increased risk. On the other hand, Avidan et al. incorporated insomnia status following treatment into their analysis and suggested that untreated or not well-treated insomnia may play an important role in elevating risk for fall and fracture. They reported the risk for fall was not increased in subjects with inactive insomnia under hypnotic treatment. They further hypothesized that treating insomnia with short-acting, non-benzodiazepine drugs may reduce the risk for subsequent falls [19]. Nevertheless, this hypothesis still needs to be further verified.

The mechanism underlying the increased risk for injuries in the patients treated with zolpidem was not explored in present study. Several mechanisms are hypothesized to elevate the risk for injury in this population. It is reasonable to propose this association may be partially attributable to the adverse effects of zolpidem in disturbing central nervous system function. This may be particularly the case among the elderly and subjects who used zolpidem inappropriately as zolpidem abuse has been reported to be associated with risky driving behavior [6]. Co-existing psychiatric and psychological diseases may also be associated with high risk behaviors, intentional injury, and self-inflicted injury.

Zolpidem associated parasomnia is another potential cause of injury. Such behavior may lead to additional damage to both patients themselves and other persons. Furthermore, the mechanisms for specific injuries may be informative in understanding the cause of injuries in the patients treated with zolpidem. For example, committing suicide by wrist cutting may be a cause of injury to the blood vessel. Nevertheless, the mechanisms underlying the association between zolpidem and injury may be very complex and heterogeneous.

Several limitations of our study should be addressed. First, the compliance and usage patterns of zolpidem could not be determined from our database. Therefore our study may have misestimated the cumulative dose of zolpidem in patients who used the drug irregularly. In addition, some patients may have misused the drug and self-adjusted their zolpidem dose or taken it at inappropriate times. These behaviors can elevate the risk of daytime side effects and increase the risk for associated injury. Second, the co-morbidity status of the subjects can also affect the risk for injury. Fragility and multiple co-morbidities can increase the risk for falls and subsequent injury, particularly in the elderly [20]. The occurrence of an injury is unsurprisingly multi-factorial. Both individual factors including behavior, comorbidity, fragility, and environmental factors are all involved in injury occurrence [21]. Therefore, the selection of appropriate adjusting co-variables in the estimation of the overall risk for injury is a complex decision.

Third, several medications, such as antidepressants and other psychoactive medications, have been recognized to increase the risk of falls and fracture [22], [23]. However, we did not take co-prescribed medications into consideration in the model utilized in this study as they have strong co-linearity with the co-morbidities. In order to be eligible to take a medication a subject is required to have an appropriate diagnosis. If a subject had received a diagnosis, it would have been accounted for when adjusting for co-morbidities. If a subject did not receive a diagnosis, they definitely would not be taking a medication. This situation caused strong co-linearity and precluded our analysis of co-used medications. In order to better adjust for pre-existing conditions we have now adjusted for the co-morbidities included in the Elixhauser Co-morbidity Index (30 co-morbidity measures).

Fourth, the detailed cause of injury is not recorded in our database. Therefore, the differentiation between non-intentional and intentional injury, including suicidal attempts and self-inflicted injuries, cannot be determined from our database. This information is useful in clarifying the mechanisms underpinning the association of zolpidem with injury.

Fifth, the existence and duration of insomnia in the present study could not be determined. In addition, the diagnostic criteria for insomnia may not be uniform among physicians. These factors may result in heterogeneity of the study population. Finally, potentially confounding variables such as body mass index, alcohol consumption, and level of physical activity were not recorded in our database. These factors also play a modifying or mediating role in injury occurrence [24].

### Conclusion

We found that patients treated with zolpidem had higher risk than a matched comparison cohort of a wide spectrum of injuries. We also found the risk for severe forms of injuries, such as intracranial injuries, injuries to the internal organs, nerves and spinal cord were also increased in these patients. In addition, patients with prolonged zolpidem (>30 days) use were suggested to be at a higher risk for injury than patients only taking zolpidem for a short period of time. Careful assessment and precaution is advised to prevent injury in this population.
